# Enhanced Effectivity of an ALK5-Inhibitor after Cell-Specific Delivery to Hepatic Stellate Cells in Mice with Liver Injury

**DOI:** 10.1371/journal.pone.0056442

**Published:** 2013-02-18

**Authors:** Marike Marjolijn van Beuge, Jai Prakash, Marie Lacombe, Eduard Post, Catharina Reker-Smit, Leonie Beljaars, Klaas Poelstra

**Affiliations:** 1 Department of Pharmacokinetics, Toxicology and Targeting, University of Groningen, Groningen, The Netherlands; 2 Kreatech Diagnostics, Amsterdam, The Netherlands; Institute of Hepatology, Foundation for Liver Research, United Kingdom

## Abstract

Transforming growth factor-β (TGF-β) is a major pro-fibrotic cytokine, causing the overproduction of extracellular matrix molecules in many fibrotic diseases. Inhibition of its type-I receptor (ALK5) has been shown to effectively inhibit fibrosis in animal models. However, apart from its pro-fibrotic effects, TGF-β also has a regulatory role in the immune system and influences tumorigenesis, which limits the use of inhibitors. We therefore explored the cell-specific delivery of an ALK5-inhibitor to hepatic stellate cells, a key cell in the development of liver fibrosis. We synthesized a conjugate of the ALK5-inhibitor LY-364947 coupled to mannose-6-phosphate human serum albumin (M6PHSA), which binds to the insulin-like growth factor II receptor on activated HSC. The effectivity of the conjugate was evaluated in primary HSC and in an acute liver injury model in mice. *In vitro*, the free drug and the conjugate significantly inhibited fibrotic markers in HSC. In hepatocytes, TGF-β-dependent signaling was inhibited by free drug, but not by the conjugate, thus showing its cell-specificity. *In vivo*, the conjugate localized in desmin-positive cells in the liver and not in hepatocytes or immune cells. In the acute liver injury model in mice, the conjugate reduced fibrogenic markers and collagen deposition more effectively than free drug. We conclude that we can specifically deliver an ALK5-inhibitor to HSC using the M6PHSA carrier and that this targeted drug reduces fibrogenic parameters *in vivo*, without affecting other cell-types.

## Introduction

Transforming growth factor β (TGF-β) is a major pro-fibrogenic cytokine during liver fibrosis, playing an important role in various cellular processes such as cell proliferation, apoptosis, differentiation, migration, stimulation of extracellular matrix (ECM) synthesis, and downregulation of ECM degradation [1]. TGF-β binds to the TGF-β type-II receptor on the cell surface, which then heterotetramerizes with a type-I receptor, in most cases activin-like kinase 5 (ALK5) [2]. The signal via ALK5 is further propagated by phosphorylation of Smad 2/3 transcription factors. The translocation of phosphorylated Smad 2/3 to the nucleus, together with co-transcription factors, leads to transcription of pro-fibrotic genes [1]. Additionally, TGF-β activates many other pathways which may have pro-fibrotic effects [3].

The inhibition of the TGF-β pathway directly by small molecule inhibitors or via indirect strategies has been investigated as a potential strategy for the treatment of fibrotic diseases. Since TGF-β is a key regulator of fibrogenesis, it is an attractive target for anti-fibrotic treatments. In animal models for liver fibrosis and pulmonary fibrosis, inhibition of the TGF-β pathway has been shown to have anti-fibrotic effects [4], [5], [6], reducing extracellular matrix deposition and pro-fibrotic cytokines.

Although inhibition of the TGF-β receptor seems a rational strategy, it might cause serious side-effects, since TGF-β signaling also plays an important role in tumor suppression, immune regulation and many physiological functions involving cell differentiation [7]. For this reason we propose to deliver the ALK5-inhibitor specifically to the key fibrogenic cells, in this case the HSC in the liver. By coupling it to mannose-6-phosphate human serum albumin (M6PHSA), specific uptake of the drug by activated HSC occurs [8].

During liver fibrosis, hepatic stellate cells (HSC) are primarily activated by TGF-β in addition to other pro-fibrotic cytokines. Upon activation, HSC proliferate and differentiate into myofibroblasts which secrete several extracellular matrix constituents, including collagens, laminin and fibronectin, [9], [10]. Furthermore, TGF-β induces other pro-fibrotic factors, such as connective tissue growth factor (CTGF) [11], which in turn enhances the effects of TGF-β. All together, the activated HSC are the key cells involved in the progression of liver fibrosis.

During activation of HSC, the mannose-6-phosphate/insulin-like growth factor II (M6P/IGFII) receptor is highly upregulated on the plasma membrane of these cells [12], [13]. The M6PHSA-conjugate binds to this receptor and is taken up into the cell through endocytosis [8]. The multifunctional M6P/IGFII-receptor traffics between the Golgi and the endosomal-lysosomal network and also shuttles to the plasma membrane [14]. A drug coupled to the carrier protein will be therefore taken up preferentially by the activated HSC.

We hypothesize that coupling of an ALK5-inhibitor to M6PHSA will increase its uptake in HSC and prevent unwanted effects in hepatocytes and immune cells. We examined this approach *in vitro* and *in vivo* to establish whether cell-specific inhibition of ALK5 in HSC can be a potential strategy to treat liver fibrosis. We established the characteristics of the conjugate and found *in vitro* HSC-specific effects. *In vivo*, two different doses of conjugate gave specific effects in an acute model of CCl_4_-induced liver injury, where our target receptor was upregulated, with an increase in effect compared to the free drug.

## Materials and Methods

### Materials

ALK5-inhibitor 3-(Pyridin-2-yl)-4-(4-quinonyl)]-1H-pyrazole, also known as LY-364947, was purchased from Calbiochem (Merck Chemicals, Darmstadt, Germany). Recombinant human TGF-β1 was purchased from Roche Diagnostics (Mannheim, Germany). Primary antibodies used are mouse anti-α-smooth muscle actin, mouse anti-β-actin, mouse anti-fibronectin and mouse anti-desmin (Sigma, St.Louis, MO), rat anti-CD68 (AbD Serotec, Oxford, UK), rat anti-CD31 (BD Pharmingen, San Diego, CA), goat anti-human serum albumin and rabbit anti-human serum albumin (Cappel, Zoetermeer, Netherlands), goat anti-collagen I and goat anti-collagen III (Southern Biotech, Birmingham, AL), rabbit anti-phosphorylated Smad 2 (Ser 465/467) (Cell Signaling, Beverly, MA), goat anti-Smad 2 (S-20) and goat anti-CTGF (L-20) (both Santa Cruz Biotechnology, Santa Cruz, CA). Species-specific HRP or TRITC-coupled secondary antibodies were purchased from DAKO (Glostrup, Denmark).

### Synthesis of LY-364947-ULS-M6PHSA (LY-conjugate)

The Universal Linkage System (ULS™) - developed by Kreatech Diagnostics, Amsterdam, The Netherlands - is a platinum based linkage technology which facilitates the coupling of molecules directly to each other through the formation of a coordinative bond. The ULS™ technology has been proven to have important applications in the area of genomics, proteomics, diagnostics, and therapeutics. The inhibitor was conjugated to the ULS-linker as previously reported [15]. M6P_28_HSA was synthesized and characterized as described elsewhere [16]. ULS – LY-364947 (12,5 µmol) was subsequently reacted with M6PHSA (0,83 µmol) in tricine buffer at 37°C. After overnight reaction the conjugate was extensively dialyzed and purified.

### Characterization of LY-conjugate

The amount of LY-364947 coupled to M6PHSA was determined by HPLC analysis after chemically displacing the drug from the carrier by overnight incubation with 200 mM sodium dithiocarbamate at 80°C. M6PHSA protein concentration was determined by Lowry assay (Bio-Rad, Hercules, CA). The stability of the LY-conjugate was also determined after 1 or 2 freeze-thaw cycles. HPLC analysis was performed on a C_18_ reversed-phase SunFire column (Waters, Milford, MA) using a mobile phase of water-acetonitrile-trifluoroacetic acid (91∶9∶0.1, vol/vol/vol; pH 2.0) at a flow-rate of 1 ml/min. LY-364947 was detected at 320 nm and eluted after circa 6.0 minutes. Quantitation of the drug levels was done by analyzing peak areas using calibration curves.

### Cell culture

HSC were isolated from the livers of male Wistar rats (>500 g, Harlan, Netherlands) according to previously published methods [17]. After isolation, HSC were cultured on plastic for 7 days until activation, and then used for experiments. HepG2 cells were cultured in DMEM (Gibco, Invitrogen, Carlsbad, CA) containing 10% fetal calf serum and penicillin/streptomycin. For conjugate binding assays cells were incubated for 2 h at 37°C with 0.1 mg/ml of the LY-conjugate, pre-incubation with antibody was 30 min.

For the Smad-reporter assay Mv1Lu cells were used, stably transfected with a SBE-Luc reporter plasmid as described previously [18], [19]. These cells express the M6P/IGFII receptor, and the Mv1Lu model is a well-defined system to assess the effect of interventions on the TGF/Smad signaling pathway. Luciferase assay was performed using a Luciferase Assay System (Promega, Madison, WI) according to the manufacturer's instructions. Cell viability was determined by fluorescent alamarBlue assay (Serotec, Oxford, UK), according to the manufacturer's instructions.

### Animal experiments

All protocols for animal experiments were approved by the Institutional Animal Care and Use Committee of the University of Groningen, the Netherlands (permit number 5232C). All animals were purchased from Harlan (Zeist, Netherlands) and kept at 12 h light/12 h dark cycles with ad libitum chow and water.

### CCl_4_ -induced acute liver injury

For *in vivo* studies in the acute (72 h) model, male C57/Bl6 mice (20–22 g) received a single intraperitoneal injection of 1 ml/kg CCl_4_ diluted in olive oil. Control mice received only olive oil. The mice were then divided into 5 groups (4 animals per group): 1) CCl_4_+vehicle (PBS), 2) CCl_4_+LY-conjugate (equivalent to 650 µg/kg/day LY-364947), 3) CCl_4_+LY-conjugate (equivalent to 1300 µg/kg/day LY-364947), 4) CCl_4_+LY-364947 (650 µg/kg/day), 5) CCl_4_+LY-364947 (1300 µg/kg/day). All treatment groups received 2 i.v. injections, 24 and 48 h after CCl_4_ and were sacrificed 24 h after the last injection. A blood cell count was performed and serum markers were determined according to standard clinical procedures at the University Medical Center Groningen.

### Real time RT- PCR

Total RNA was isolated from HSC using the Absolutely RNA Microprep Kit (Stratagene, La Jolla, CA) and from tissue homogenates using the RNeasy Mini kit (Qiagen, Hilden, Germany). The amount of RNA was determined using a NanoDrop UV-detector (Nano Drop Technologies, Wilmington, DE). Synthesis of cDNA was performed using random primers; for isolated HSCs, the Superscript III first-strand synthesis kit (Invitrogen, Carlsbad, CA) was used, while for tissue samples AMV Reverse Transcriptase (Promega, Madison, WI) was used. All primers were purchased from Sigma Genosys (Haverhill, UK). Gene expression levels were measured by real-time quantitative PCR on an ABI 7900HT apparatus (Applied Biosystems, Foster City, CA) with SYBR-Green PCR Master Mix (Applied Biosystems). The formation of single products was confirmed by analyzing the dissociation step at the end of each PCR reaction. Data were analyzed using the SDS 2.3 software program (Applied Biosystems). The relative amount of product was calculated using a calibration curve, normalizing for the expression of the household gene GAPDH and related to the control treatment.

### Western blot

Collagen I expression in the livers of CCl_4_-mice and Smad2 phosphorylation in HepG2 and HSC cells were determined using Western blot analysis. Fifty µg of protein from each sample was applied on a SDS-PAGE gel en the proteins were transferred to a polyvinylidene fluoride membrane electrophoretically. Membranes were blocked with 5% nonfat milk in Tris-buffered saline containing 0.5% Tween-20 and then incubated with primary antibody overnight at 4°C. After washing horseradish peroxidase-conjugated secondary antibody was applied for 2 h. Protein bands were developed with ECL detection reagent (Perkin-Elmer Life Sciences, Boston, MA) and quantified using the GeneSnap program (SynGene, Synoptics, Cambridge, UK). Collagen I expression was normalized to β-actin levels and Smad phosphorylation to total Smad2 levels.

### Immunohistochemistry

Immunohistochemistry and immunofluorescence were performed on 4 µm cryo- and paraffin sections. Immunocytochemistry was performed on cells fixated in methanol-acetone. Stainings were visualized using 3, 3′-diamino-benzidine tetrahydrochloride or 3-amino-9-ethylcarbazole. When necessary, immunofluorescent staining in sections was visualized using a M.O.M.-kit (Vector Laboratories, Burlingame, CA) according to the manufacturer's instructions. Nuclei were counterstained with Mayer's hematoxylin or DAPI. Immunohistochemical stainings were quantitated using the Cell D computer program (Olympus, Hamburg, Germany), according to the parameters described in the respective figure legends.

### Statistical analysis

Results are expressed as the mean ± SD, unless otherwise specified. Statistical analyses were performed using Student's t test or one-way ANOVA with post-hoc Bonferroni test. p<0.05 was considered as the minimum level of significance.

## Results

### Characterization of LY- conjugate

**Figure 1:**
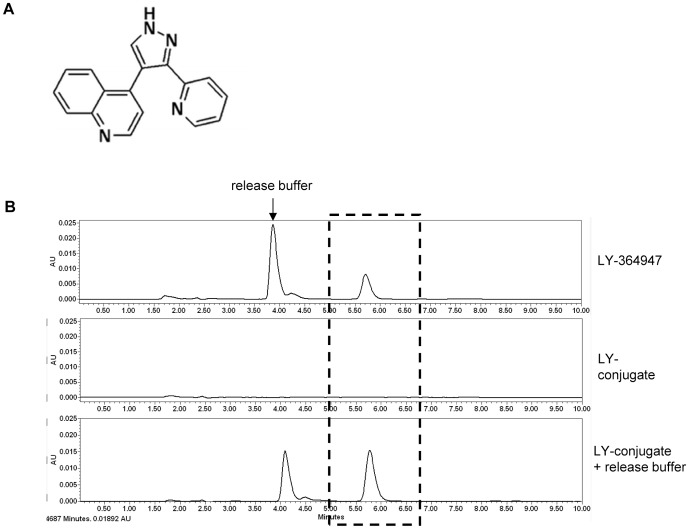
Synthesis and characterization of LY-364947-ULS-M6PHSA. (**A**) Structure of LY-364947. (**B**) HPLC analysis of LY-conjugate: free LY-364947 (upper panel), LY-conjugate without treatment to release drug (middle panel) and LY-conjugate after treatment with 200 mM sodium dithiocarbamate to release the drug from the carrier (lower panel).

The ALK5-inhibitor LY-364947 was successfully conjugated to M6PHSA using the ULS linker. To determine the amount of LY-364947 (Fig. 1A) in the LY-M6PHSA conjugate, the concentrations of both protein and drug in the conjugate were determined using a protein assay and the HPLC method, respectively. The average ratio of drug to protein was 10 ∶ 1 and there were no major differences between different batches synthesized in the course of this study. HPLC analysis also showed that the drug can be released from the conjugate in release buffer containing di-thiocarbamate (Fig. 1B), but there was no release after repeated freeze-thawing (results not shown) indicating the stability of the conjugate. The release of drug by thiol (– SH) containing groups indicates that the drug can be displaced from the carrier by intracellular components like glutathione.

### *In vitro* anti-fibrotic effects of the LY-conjugate

**Figure 2:**
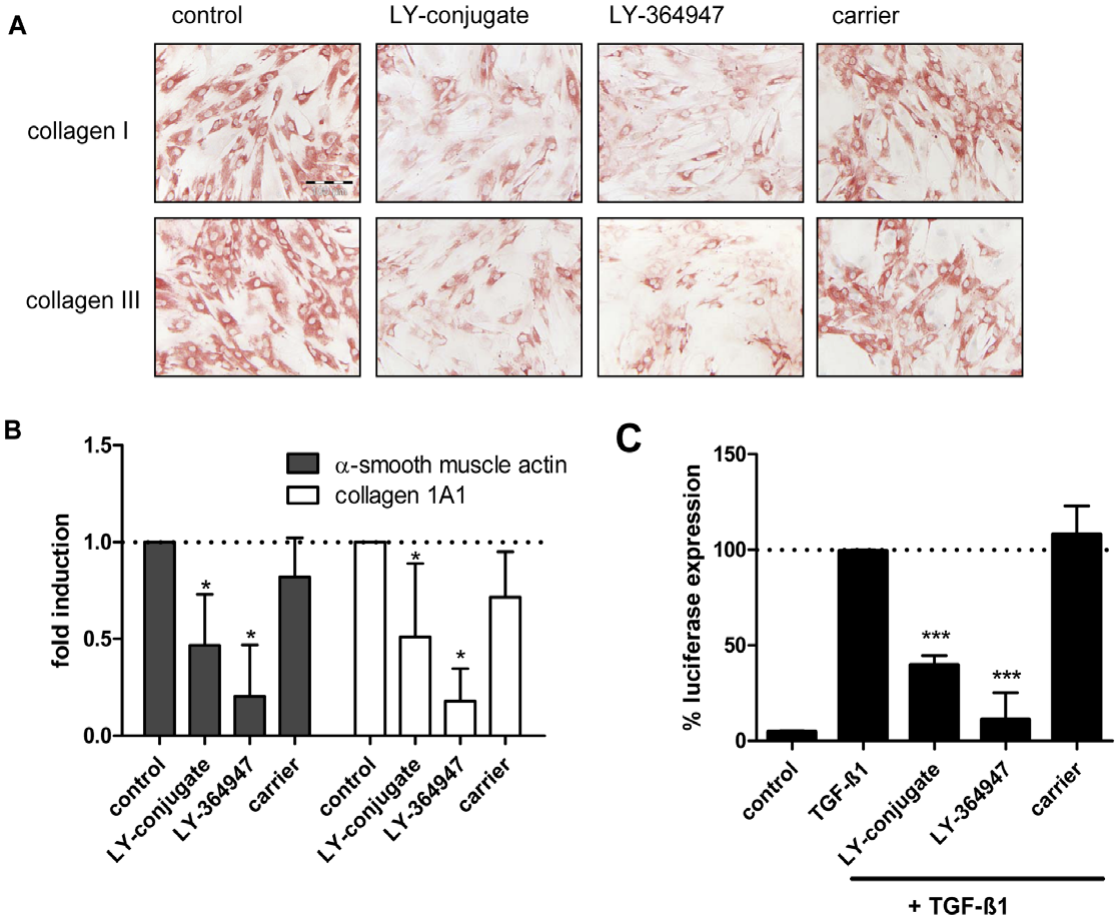
In vitro effects of LY-conjugate. (**A**) Collagen deposition by HSC incubated with LY-conjugate (equivalent to 10 µM free drug), LY-364947 (10 µM) or carrier (molar equivalent). Cells were stained for collagen I and III. Scale bar denotes 100 µm. (**B**) Effect of LY-conjugate (equivalent to 10 µM free drug), LY-364947 (10 µM) and carrier (molar equivalent) on the fibrotic markers α-SMA and collagen 1A1 in isolated rat HSCs after 48 h incubation. * p<0.05 vs. control by Student's t-test. (**C**) LY-conjugate and LY-364947 reduce luciferase expression in mink epithelial cells with a SBE-Luc reporter. *** p<0.001 vs. TGF-β1 by Student's t-test.

In order to examine the anti-fibrotic activity of the LY-conjugate, we used primary rat HSC that are spontaneously activated after 7 days of culture. We found that treatment with LY-conjugate resulted in a profound inhibition of both collagen type I and III deposition (Fig. 2A). Parallel experiments demonstrated that at the mRNA level, two key markers of fibrosis, i.e. α-smooth muscle actin (α-SMA) and collagen 1A1, were also significantly reduced both by the free drug and the conjugated drug (Fig. 2B). The free drug was more potent in this *in vitro* system, presumably because the free drug enters into the cells rapidly, providing high intracellular drug levels, in contrast to the targeted construct which enters through a receptor-mediated endocytosis process.

### Effects of LY-conjugate on TGF-β signaling *in vitro*

We also assessed whether conjugation of LY-364947 to the carrier protein altered the inhibitory effects mediated via the TGF pathway. To that end, we examined the effect of the conjugate on the expression of luciferase in mink cells that were stably transfected with the luciferase reporter gene. The latter is controlled by a promoter containing Smad-binding elements (SBE), and is thus responsive to p-Smad 2/3 in combination with Smad4. Both free LY-364947 and the conjugate significantly inhibited TGF-β1-induced Smad signaling, measured as luciferase activity (Fig. 2C), whereas the carrier itself did not have any effect. The reduction in luciferase expression was not due to toxicity of the drug or the conjugate, as measured by AlamarBlue assay (data not shown).

### Specificity of the LY-conjugate *in vitro*

**Figure 3:**
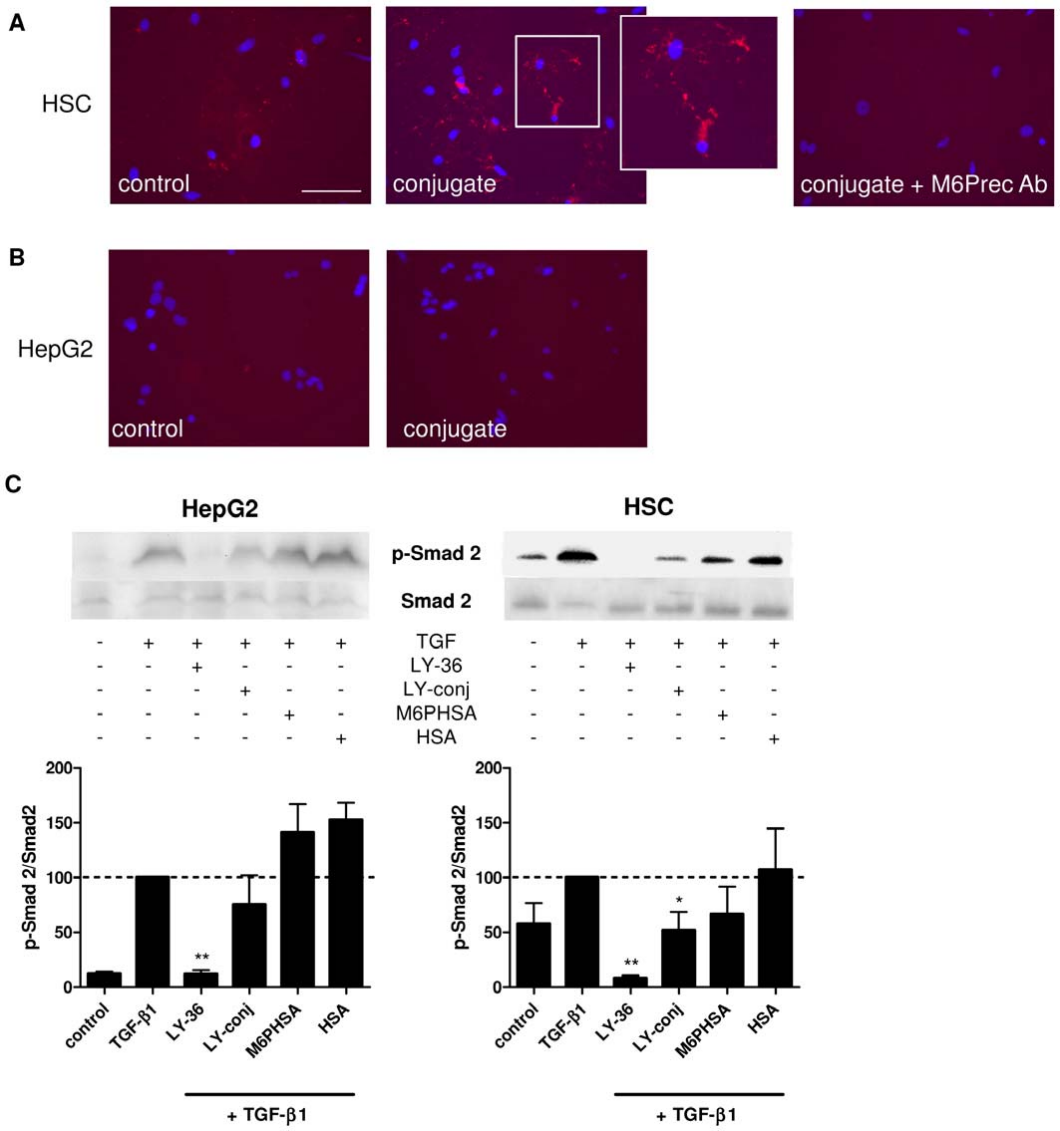
Binding of LY-conjugate and effect in hepatocytes. (**A**) HSA staining showing the binding of LY-conjugate to HSC: control cells (left), LY-conjugate-incubated cells (middle), and LY-conjugate-incubated cells pretreated with a M6P/IGFII receptor-specific antibody (right). Note that blocking of the receptor reduces binding of the conjugate to the cells. Scale bar denotes 100 µm. (**B**) HSA staining showing the binding of LY-conjugate to HepG2 cells: control cells (left), LY-conjugate incubated cells (right). (**C**) TGF-β1-induced phosphorylation of Smad2 in HepG2 cells and in HSC after incubation with LY-364947, conjugate, carrier or HSA. Representative western blots and quantitative analysis of blot density (n = 3), * p<0,05 vs. TGF-β1, ** p<0.01 vs. TGF-β1 by Student's t-test.

The binding of the conjugate to primary rat HSC was examined by immunofluorescent anti-HSA staining on cells incubated with the conjugate. The conjugate bound to activated rat HSC and this binding was strongly reduced by pre-incubation of the cells with a specific antibody against the M6P/IGFII-receptor, confirming that the conjugate binds specifically to the target receptor (Fig. 3A).

The specificity of the conjugate was also determined by examining the effect of both free LY364947 and conjugate on HepG2 hepatocytes. The conjugate did not bind to these cells (Fig. 3B). In addition to this, we found that free drug could almost completely inhibit TGF-β-induced Smad phosphorylation (90% reduction) in hepatocytes, whereas the conjugate did not have a significant effect on phosphorylation levels in these cells. In HSC in contrast, which do express the M6P/IGFII-receptor abundantly, both the free inhibitor and the conjugate significantly inhibited TGF-β1-induced Smad phosphorylation (Fig. 3C).

### Biodistribution of the LY-conjugate *in vivo*

**Figure 4:**
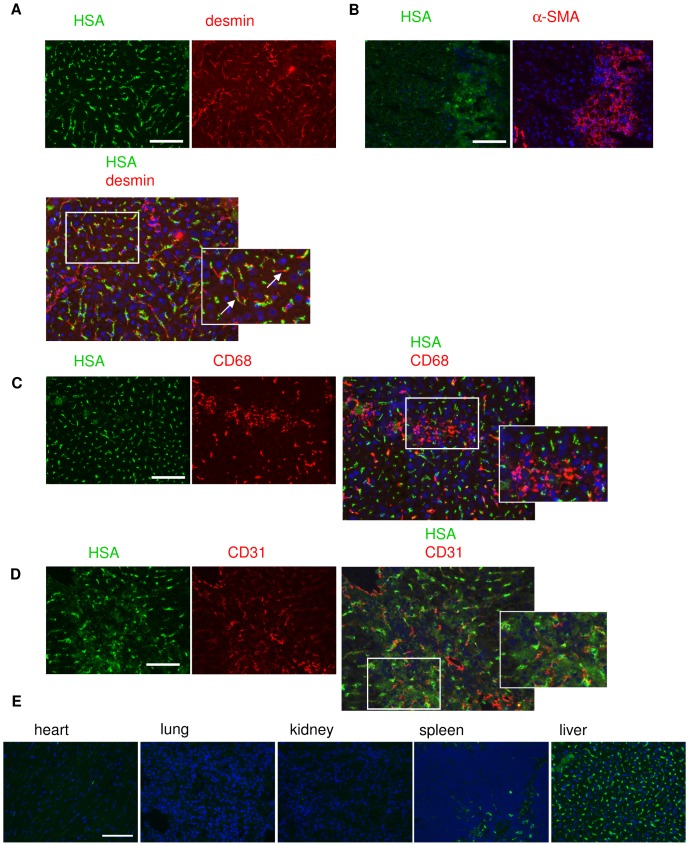
Localization of LY-conjugate in liver. Representative photographs of LY-conjugate uptake in mouse livers 60 min after injection of LY-conjugate in CCl_4_-treated mice. (**A**) Double staining for HSA (green) and the HSC marker desmin (red). (**B**) Consecutive sections stained for HSA (green) and the activated HSC marker α-smooth muscle actin (red). (**C**) Double staining for HSA (green) and the Kupffer cell marker CD68 (red) (**D**) Double staining for HSA (green) and the endothelial cell marker CD31 (red) (**E**) LY-conjugate organ localization as determined by HSA-staining (green) in heart, kidney, lung, spleen and liver 60 min after injection of LY-conjugate in CCl_4_-treated mice. Scale bar denotes 100 µm in all pictures.

We furthermore examined whether the LY-conjugate accumulated into the target cells *in vivo*. The intrahepatic distribution of the LY-conjugate was determined 60 minutes after systemic administration in CCl_4_-treated mice. The HSA-staining (green) localized to desmin-positive HSC (red) in the liver (Fig. 4A). HSA staining was also found in the same area as α-smooth muscle actin-positive cells, a marker for activated HSC (Fig. 4B). There was no co-localization of HSA (green) and CD68-positive (Kupffer) cells (red) (Fig. 4C) or HSA (green) and CD31-positive endothelial cells (Fig. 4D). No staining for HSA was observed at all in hepatocytes (Fig. 4A–D). To demonstrate organ-specificity, we performed an anti-HSA-staining in other major organs. The staining for HSA showed no accumulation of LY-conjugate in heart, kidney or lung 60 min after injection, whereas there was a small amount present in the spleen (Fig. 4E), where it was confined to the marginal zone. No staining was found in the red or white pulp.

### Effects of the LY-conjugate *in vivo*

**Figure 5:**
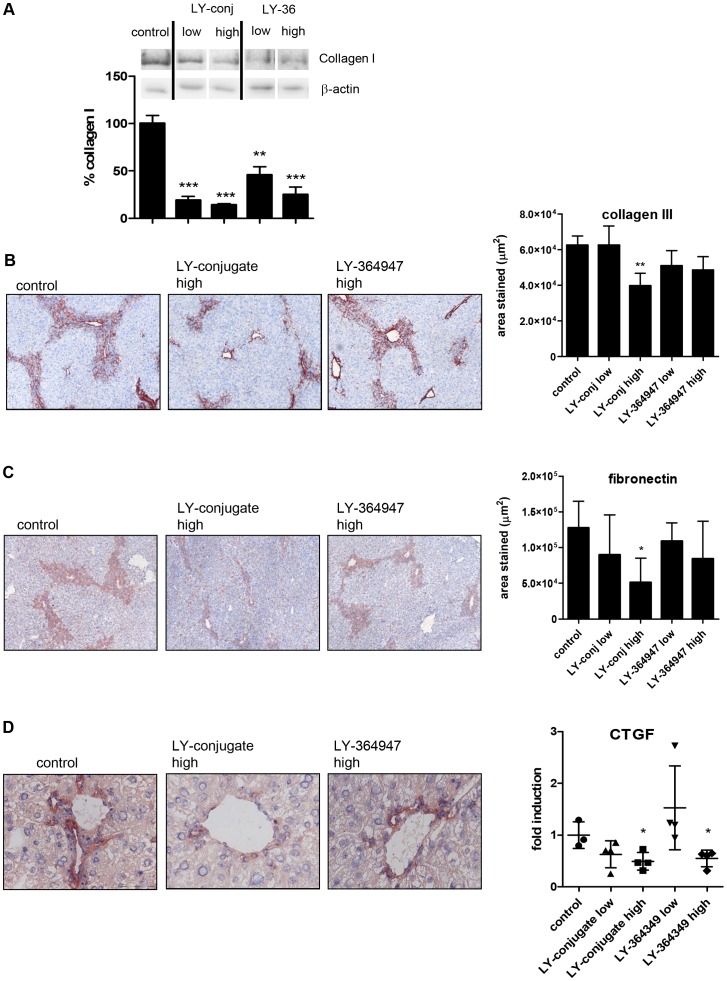
LY-conjugate reduces collagen deposition in livers of CCl_4_ mice. (**A**) Western blot analysis of collagen I expression in livers of C57Bl/6 mice, after one injection of CCl_4_ and treated with vehicle (PBS), LY-conjugate (low and high dose) or LY-364947 (low and high dose). Figures show representative blots and quantitative analysis of western blots, n = 3–4 per group. * p<0.05 vs. CCl_4_-PBS by one way ANOVA with Bonferroni post-hoc test. (**B**) Representative pictures and quantitation of immunohistochemical stainings for collagen III on liver sections of C57Bl/6 mice, after one injection of CCl_4_ and treated with vehicle (PBS), LY-conjugate (low and high dose) or LY-364947 (low and high dose). Stainings were quantitated using the Cell D software, calculating the total stained area in 18–24 fields per section at 100× magnification as a percentage of the stained area in the control CCl_4_ sections. Data shown are the mean of 3–4 animals per group. * p<0.05 vs. CCl_4_-PBS by Student's t-test. (**C**) Representative pictures and quantitation of immunohistochemical stainings for fibronectin on liver sections of C57Bl/6 mice, after one injection of CCl_4_ and treated with vehicle (PBS), LY-conjugate (low and high dose) or LY-364947 (low and high dose). Quantitation of the relative area stained positive for fibronectin was performed as described above. (**D**) Representative pictures of immunohistochemical staining for connective tissue growth factor on livers of C57Bl/6 mice, after one injection of CCl_4_ and treated with vehicle (PBS), LY-conjugate (high dose) or LY-364947 (high dose). Original magnification 400×. Expression levels of connective tissue growth factor mRNA in livers of C57Bl/6 mice, after one injection of CCl_4_ and treated with vehicle (PBS), LY-conjugate (low and high dose) or LY-364947 (low and high dose). * p<0.05 vs. CCl_4_-PBS by Student's t-test.

Effectivity of the free drug and the HSC-specific conjugate was subsequently examined *in vivo* in an acute CCl_4_-induced liver injury model. We tested two different doses of unmodified LY-364947 and equimolar doses of LY-conjugate. Collagen I expression, as assessed by western blot (Fig. 5A) was significantly decreased by both the low and the high doses of conjugate, while the free drug had less effect on collagen I expression. Stainings for other extracellular matrix molecules showed further differences between free drug and conjugate. Deposition of both collagen III and fibronectin was significantly inhibited by the high dose of LY-conjugate but not by the free drug (Fig. 5B & C). These effects were not due to a difference in CCl_4_-induced damage, since all treatment groups displayed a similar amount of damage, as reflected by the PAS-staining and ALT and AST levels (data not shown). The carrier alone did not affect collagen deposition levels (Fig. S1). As the acute liver injury model also causes considerable inflammation in the liver, we examined the effects of the treatments on liver inflammatory cells, but found no effects on influx of T-cells or activation of resident macrophages (data not shown). The reduction in collagen deposition induced by our conjugate was therefore not caused by an effect on immune cells.

In order to study whether these reductions in extracellular matrix deposition coincided with a decrease in TGF-β-induced pro-fibrotic cytokines, mRNA levels of the downstream mediator CTGF were measured in mice livers. Both free LY-364947 and the HSC-specific conjugate significantly reduced CTGF mRNA levels in liver (Fig. 5D). Immunohistochemical stainings showed that CTGF expression was localized in the portal areas, and was strongly inhibited by the conjugate, but not by free LY-364947 (Fig. 5D).

## Discussion

In the present study, we demonstrated that local inhibition of TGF-β receptor type I (ALK5) in HSC using our cell-specific targeting approach *in vivo* strongly inhibits early liver fibrogenesis. Selective inhibition of ALK5 in HSC is of high interest as prolonged ALK5 inhibition elsewhere in the body or even in other cell types in the liver may induce severe adverse effects, such as cardiac problems, tumorigenesis or immune system deregulation. To achieve cell-selective delivery, we conjugated ALK5 inhibitor LY-364947 to HSC-targeting carrier M6PHSA. The LY-conjugate specifically accumulated into the target cells *in vitro* and *in vivo*. Within HSC, it blocked the ALK5 pathway and induced a strong anti-fibrogenic effect compared to equivalent doses of the free drug. These data show that selective blocking of ALK5 in HSC may result in a cell-specific therapeutic strategy.

Experimental drugs that were very effective *in vitro* or in experimental animal models have often failed to be effective in subsequent studies [20]. Exploration of drug effects after a cell-specific approach might explain why drugs fail to have the expected effect. Failure in a (pre)-clinical setting may be caused by several factors, ranging from impaired delivery in diseased tissue to dose-limiting side-effects, and these factors can be modulated by a cell-specific delivery approach. If targeted drugs are not effective, the target pathway within the target cell is of minor importance. Here we have shown that TGF-β-signaling via the ALK5-receptor in HSC is of great importance in early liver fibrogenesis.

In the current study we have demonstrated specific targeting of the LY-conjugate to HSC both *in vitro* and *in vivo*. *In vitro*, the LY-conjugate was taken up by the primary rat HSC, while blocking of the uptake with a specific antibody showed the specificity to the receptor. The conjugate was fully biologically active as it inhibited the spontaneous activation of primary HSC and it reduced Smad 2/3 signaling profoundly. Even though the conjugate was proven to be active in HSC, it did not inhibit Smad phosphorylation in the most abundantly present cell type in liver, hepatocytes, which is consistent with the fact that the conjugate did not bind to these cells. The fact that there is no effect on TGF-β signaling in hepatocytes, nor binding (*in vitro* or *in vivo*) implies a reduced risk of pro-tumorigenic effects [21] of targeted TGF-β inhibition, which is particularly relevant in hepatocytes that reside in the pro-tumorigenic fibrotic environment.

*In vivo*, specific localization of the conjugate in HSC but not in other cell types in the liver or in other organs, as demonstrated by double immunofluorescent staining, revealed the cell-specific accumulation of the conjugate. It was not possible to directly measure concentrations of this drug within the liver after treatment due to rapid metabolism of the released drug, but previous studies with a similar kinase inhibitor-conjugate have shown up to 7× higher levels of drug in the liver after treatment with conjugate as compared to treatment with free drug [22]. Furthermore, studies in kidney fibrosis using the same drug and linker have shown sustained high levels of drug within the target organ [15]. Therefore it is probable that increased efficacy of this conjugate *in vivo* is mainly due to its more favorable pharmacokinetic profile. The targeting method thus leads to a high accumulation in the target cell, increasing effectivity compared to an equimolar dose of free drug.

*In vivo* the targeted ALK5-inhibitor significantly reduced the deposition of extracellular matrix constituents, that is, collagen I and III and fibronectin. Previous experiments have shown no effects of the M6PHSA carrier in this *in vivo* model [22], so the anti-fibrotic effects are due to the targeted ALK5-inhibitor. Furthermore, the expression of the TGF-β dependent cytokine CTGF was also inhibited by the conjugate, indicating a TGF-β-inhibiting activity of the conjugate. Immunohistochemistry showed that CTGF protein expression was localized near portal tracts, most likely within the portal tract fibroblasts, as found in earlier studies [23]. This CTGF protein expression was reduced by the HSC-specific ALK5-inhibitor, but not by free drug, possibly reflecting uptake and pharmacological effects of our conjugate in the portal fibroblasts as well.

Inhibition of ALK5 has been shown to be a valuable antifibrotic strategy in animal models for fibrosis in different organs [5], [15], [24], [25], since TGF-β plays a crucial role in most fibrotic diseases. Despite the anti-fibrotic effects of TGF-β inhibitors, their use is considered unsafe due to critical side-effects [7], [21], [26], [27], [28]. Since the ALK5 is expressed ubiquitously nearly on all cell types, the inhibition of this receptor may induce many effects. Small molecule ALK5-inhibitors have been shown to cause heart valve lesions in animal models [28]. TGF-β is also known to be an important regulator of the immune system, as mice lacking TGF-β1 die from a multi-organ inflammatory syndrome [7]. Deregulation of the immune system in immune-compromised cirrhotic patients or patients with viral hepatitis poses a risk for the patient. Furthermore, TGF-β is a suppressor of early tumor growth [21]. Pre-clinical evidence suggests that inhibition of ALK5 in rats predisposed to developing renal cell carcinoma may elicit tumor development [29]. Since cirrhosis patients are at a higher risk for hepatocellular carcinoma [30] the use of ALK5-inhibitors for the treatment of liver fibrosis might therefore pose an extra risk. In this study we showed that there is no effect of the targeted conjugate in hepatocytes or uptake of the conjugate in Kupffer cells, thus reducing the chances of interfering with TGF-β signaling in other hepatic cell types.

In conclusion, we now have shown that cell-specific delivery of an ALK5-inhibitor to HSC is a novel and promising concept to be explored in further studies. It reduces activation of HSC *in vitro* and *in vivo* and inhibits extracellular matrix deposition by HSC *in vivo*, where conjugation to our drug carrier is associated with an increased effectivity of the drug in HSC. Our results also show that uptake of the drug in other organs and in neighboring parenchymal cells can be prevented by coupling to our drug carrier, preventing side-effects in pivotal cells. Thus, cell-specific targeting of drugs can be achieved while preserving or even increasing the effectivity and at the same time decreasing kinase inhibitor-specific side-effects.

## Supporting Information

Figure S1 **M6PHSA carrier does not affect collagen deposition in livers of CCl_4_ mice.** Representative pictures of immunohistochemical stainings for collagen I and collagen III on liver sections of C57Bl/6 mice, after one injection of CCl_4_ and treated with M6PHSA carrier. Original magnification 400×.
(TIF)
