# Supplementary figures and images for: Enhanced Effectivity of an ALK5-Inhibitor after Cell-Specific Delivery to Hepatic Stellate Cells in Mice with Liver Injury
Source: PLoS One. 2013 Feb 18;8(2):e56442. doi: 10.1371/journal.pone.0056442 (PMC3575413; doi:10.1371/journal.pone.0056442)

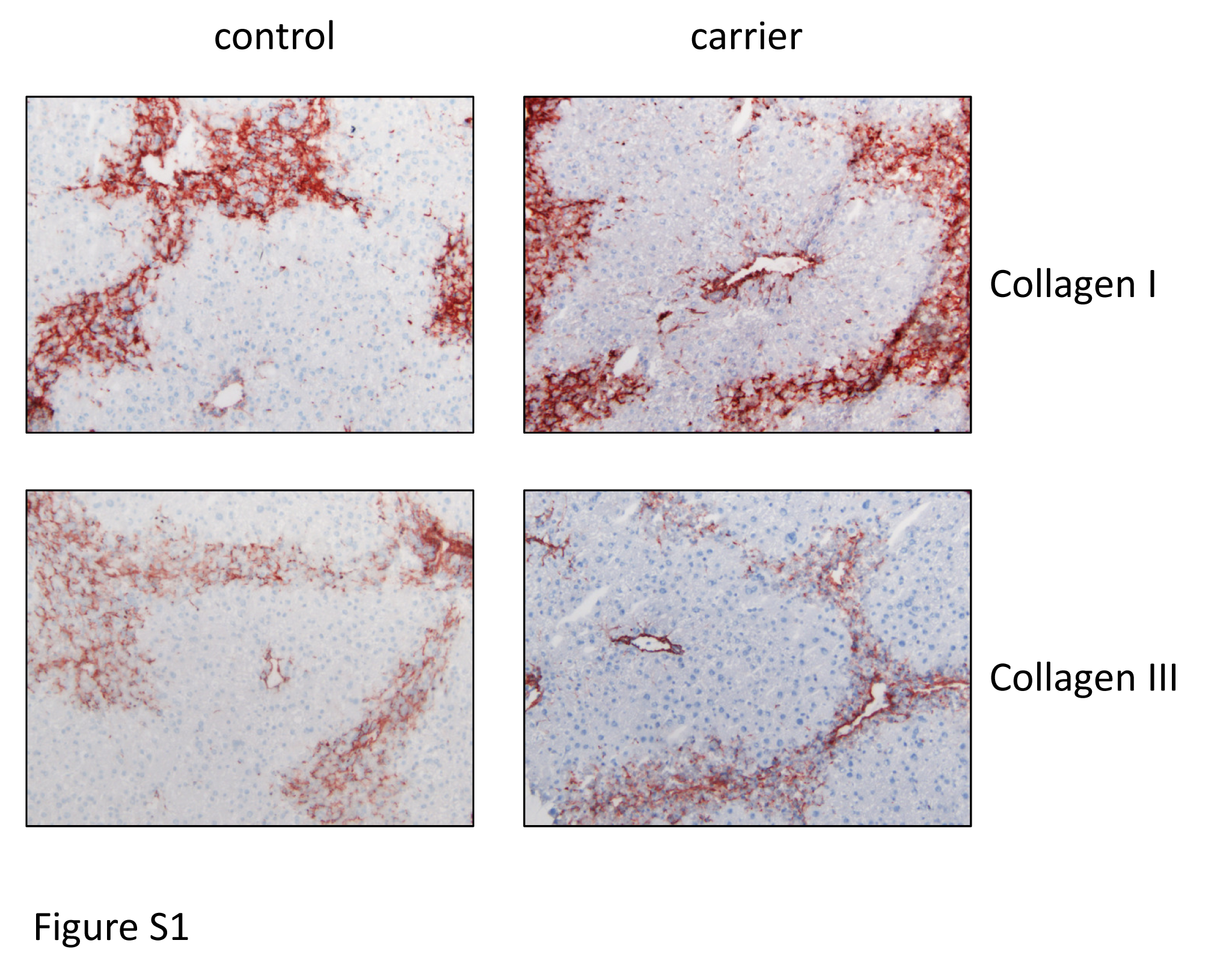

Supplement: Figure S1 — M6PHSA carrier does not affect collagen deposition in livers of CCl4 mice. Representative pictures of immunohistochemical stainings for collagen I and collagen III on liver sections of C57Bl/6 mice, after one injection of CCl4 and treated with M6PHSA carrier. Original magnification 400×. (TIF) [file pone.0056442.s001.tif]
